# Fractures of the distal femur in elderly patients: retrospective analysis of a case series treated with single or double plate

**DOI:** 10.1186/s13018-022-02944-6

**Published:** 2022-01-29

**Authors:** Dae Jin Nam, Min Seok Kim, Tae Ho Kim, Min Woo Kim, Suc Hyun Kweon

**Affiliations:** 1 Department of Orthopedic Surgery, School of Medicine, Wonkwang University, Iksan, Chunbuk Korea; 2 Department of Orthopedic Surgery, Presbyterian Medical Hospital, Jeonju, Chunbuk Korea

**Keywords:** Femur, Distal femoral fracture, Osteoporotic fracture, Double plating, Lateral parapatellar approach

## Abstract

**Introduction:**

We evaluated the radiologic and clinical outcomes of a lateral incision single plate with and a single-incision double plating in elderly patients with osteoporotic distal femoral fractures.

**Materials and methods:**

We performed a retrospective study of 82 cases of distal femoral fractures from May 2004 to June 2018. Group A consisted of 42 patients who underwent single-plate fixation. Group B consisted of 40 patients who underwent double-plate fixation. The mean patient age was 77 years (67–87 years) and 76 years (64–86 years) in groups A and B, respectively. All patients were evaluated for procedure duration, time to union, range of knee motion, Lysholm knee score, and presence of complications.

**Results:**

The average procedure time was 81 min (66–92 min) and 110 min (95–120 min) in groups A and B, respectively (*p* = 0.33). One case in group B required bone grafting after 5 months. The average time to union was 14 weeks (9–19 weeks) and 12.2 weeks (8–19 weeks) (*p* = 0.63), and the mean range of knee motion was 105° (90–125°) and 110.7° (90°–130°) (*p* = 0.37) in groups A and B, respectively. There was no significant statistical difference between the two groups in the Lysholm knee score (*p* = 0.44) and knee society score (*p* = 0.53).

**Conclusion:**

The clinical and radiological outcomes were similar in the 2 groups. In elderly patients, double plate fixation for distal femoral fractures is an useful method for several advantages such as adequate exposure, easy manipulation, anatomical reduction and stable fixation.

## Background

Distal femoral fractures account for 1% of all fractures and 4–6% of femoral fractures, and the incidence is increasing in proportion to aging [1]. These fractures occur in the elderly population with osteoporosis, similar to the pattern of fractures due to high-energy damage that occurs in young age groups. the treatment is difficult due to frequent severely comminuted fragments or intra articular frcture as well as associated soft tissue damage [2]. Supracondylar fractures can cause delayed union or nonunion that requires reoperation, regardless of the internal fixation method used during surgery, and can lead to deep infection, implant failure, and malunion [3, 4]. In particular, supracondylar fractures of the femur, especially in the elderly, mainly occur in patients with severe osteoporosis. As the distal fracture fragment is too small to obtain sufficient fixation, nonunion or irregular union and considerable bleeding occur, thereby increasing the mortality risk [5].

Accordingly, various types of internal fixation devices (anatomical plate, blade plate, dynamic condylar screw, buttress plate, intramedullary nail, etc.) have been developed and applied to surgical treatment. And the latest generation anatomical plates with variable fixed angles can represent an advantage in the aforementioned fractures with bone fragility [6]. Moreover, various biomechanical studies on internal fixation aiming to obtain optimal stability in supracondylar fractures of the femur have been conducted to date, and most of them compared the use of a locking metal plate and retrograde intramedullary nailing using a cadaver [7–11]. With most of the internal fixation devices located on the lateral side of the femur, lateral support is relatively easy to obtain; however, for the internal comminuted fracture fragment, internal reduction is unlikely to be achieved with internal fixation on the lateral side alone [12]. To achieve good results in surgical treatment, principles such as anatomical reduction of the articular surface, recovery of the axis of the lower-extremity length, rigid fixation, and early movement of the knee joint must be observed [2, 13].

The purpose of this study was to examine the usefulness of double-plate fixation by comparing its clinical and imaging results with those of internal fixation using a single plate for distal femoral fractures with comminuted fragments in patients with osteoporosis.

## Materials and methods

This study was approved by Institutional Review Board of Wonkwang University Hospital (2021-03-012).

### Subjects

**Fig. 1:**
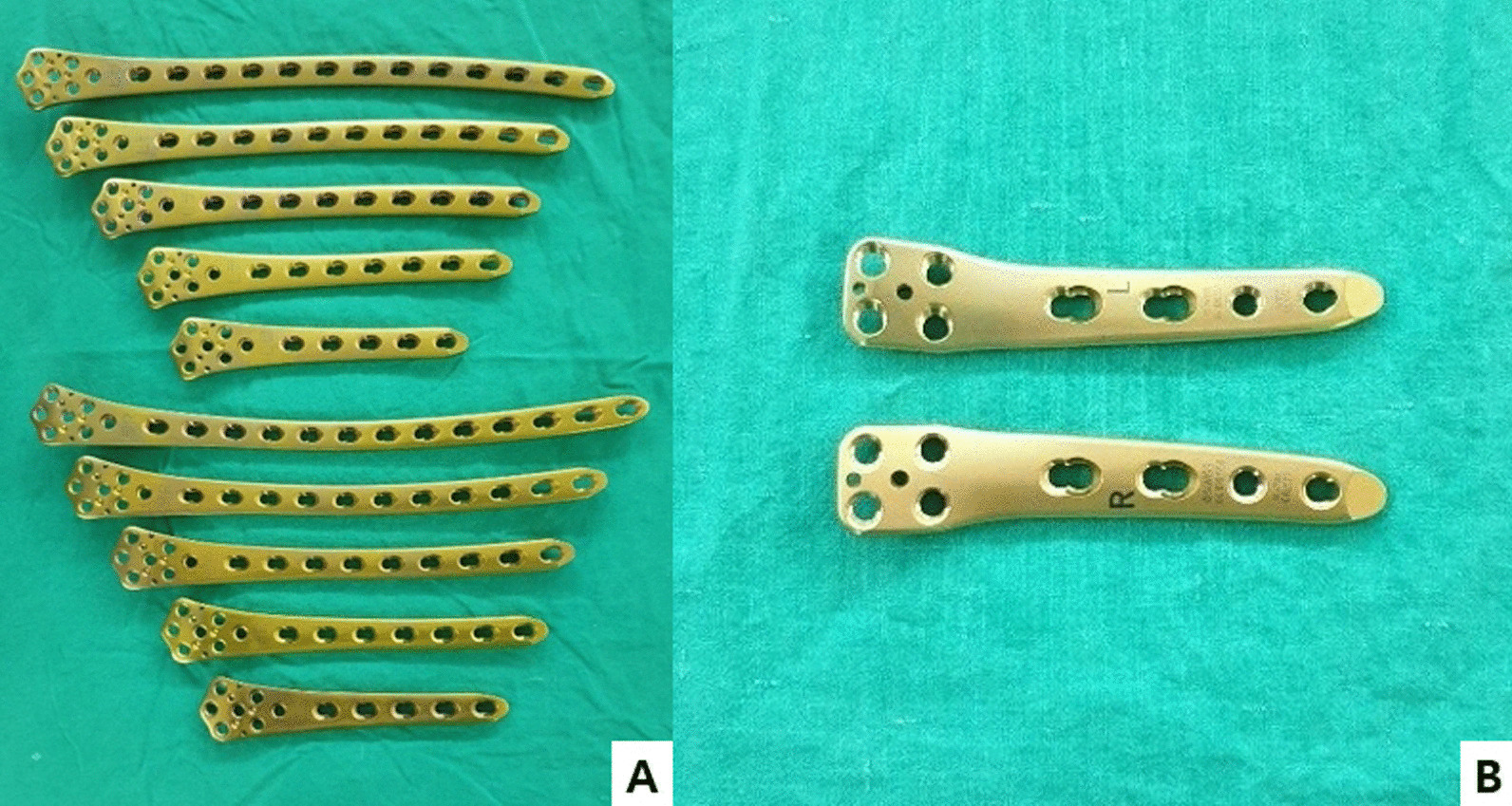
**A** Lateral plates: locking compression plates (distal femur plates). The plates are available with 5, 7, 9, 11, and 13 holes for the lateral side of the left and right femurs (Depuy Synthes approved by the AO Foundation, Switzerland and United States). **B** Medial plates: TomoFix medial distal femoral plates, available for the medial side of the left and right femurs (Depuy Synthes approved by the AO Foundation, Switzerland and United States)

From May 2004 to June 2018, patients who were followed up for at least 12 months after undergoing osteosynthesis using a metal plate were enrolled in this study. These patients had a supracondylar fracture of the femur and underwent internal fixation using a single plate (group A, 42 patients) or double plates (group B, 40 patients) (Fig. 1). The patients were retrospectively analyzed based on periodic imaging and clinical findings. The fracture pattern was identified using simple radiographic imaging and three-dimensional computed tomography, and classified according to the AO/OTA system, as follows: type A was a simple supracondylar fracture.

### Methods and rehabilitation

**Fig. 2:**
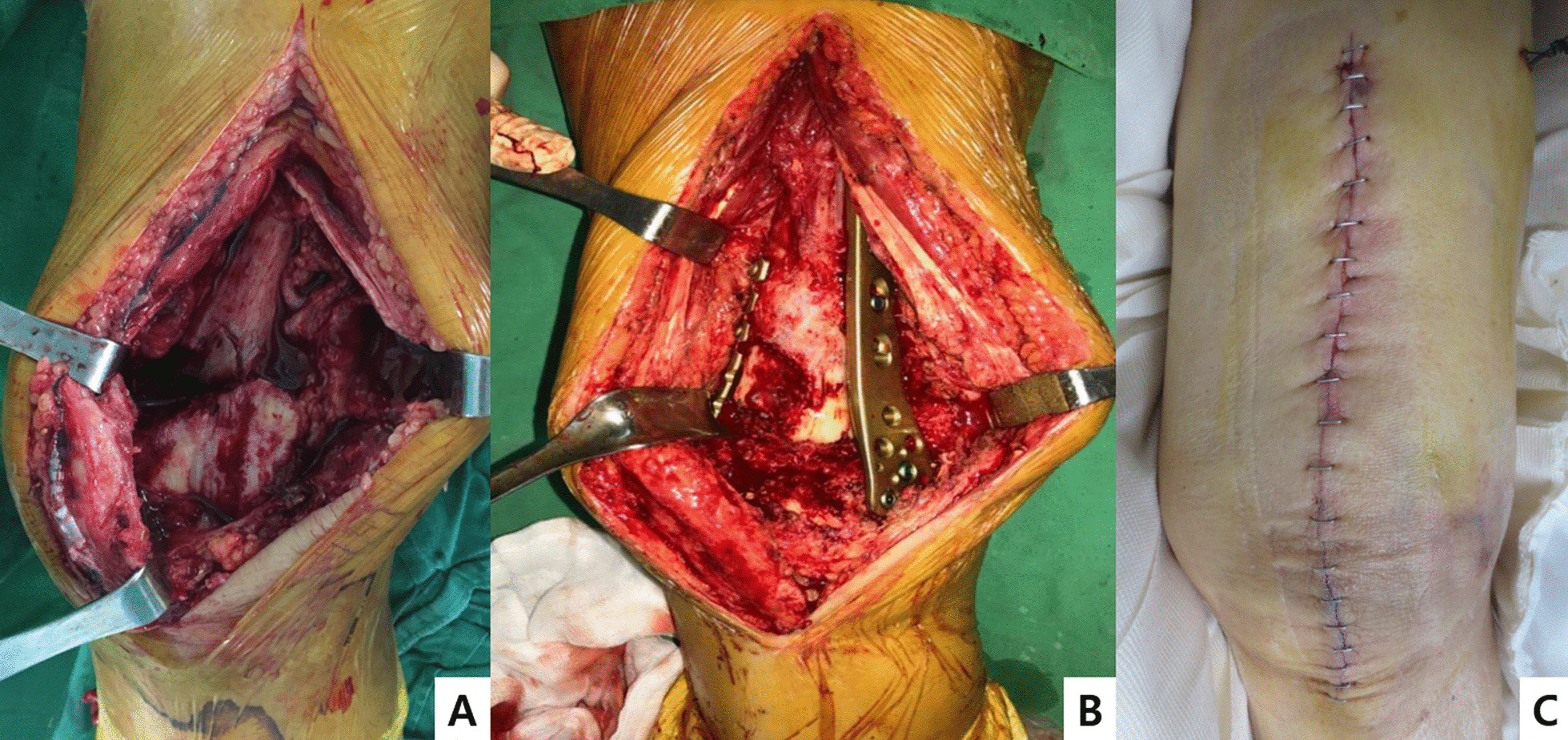
Lateral parapatellar surgical approach: the incision of the quadriceps tendon is extended toward the proximal part to sufficiently expose the fracture part in order to easily locate the medial and lateral metal plates. **A** Bone defect, **B** double plating, **C** skin incision line

**Fig. 3:**
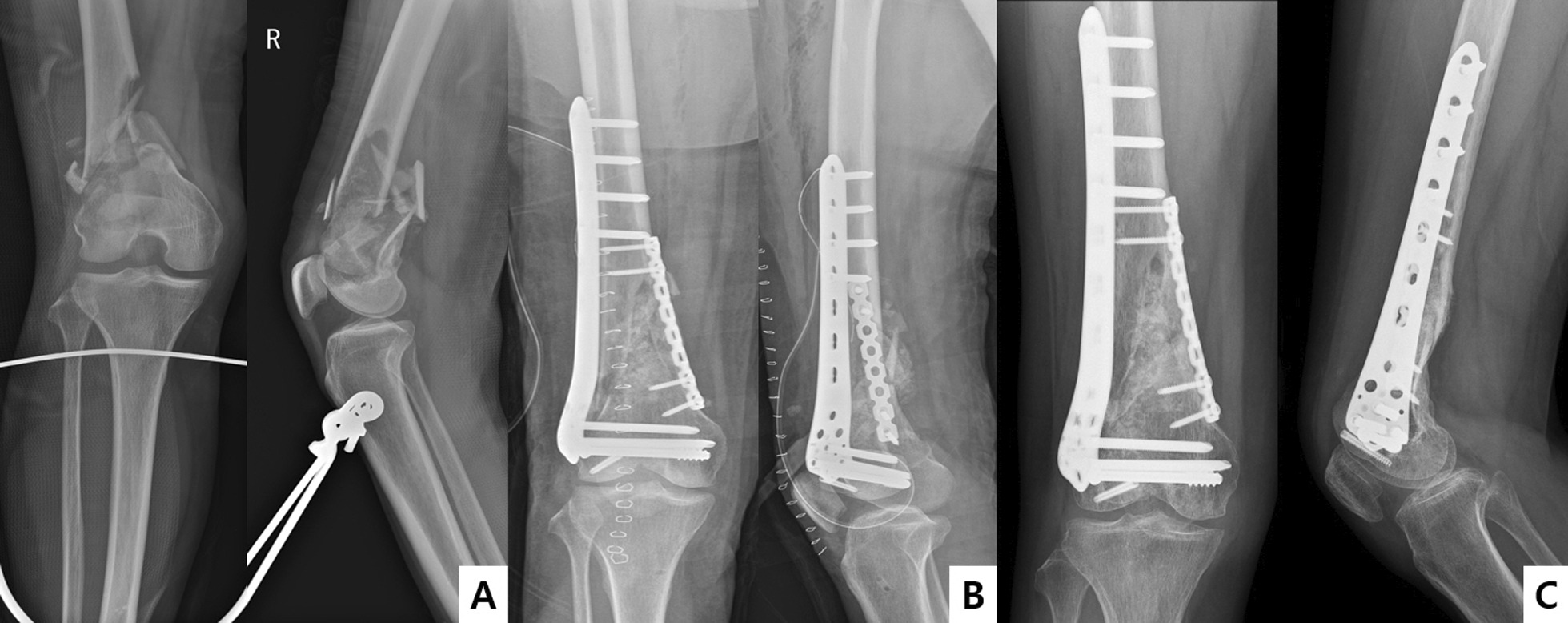
**A** Preoperative radiograph of a 70-year-old female patient showing a displaced distal femoral fracture. **B** Postoperative radiograph of the 70-year-old female patient showing the displaced distal femoral fracture. **C** Bone union was achieved at 6 months after the operation with double-plate fixation

After placing the patient in the supine position on the surgical table, the unaffected leg was lowered to allow easy identification of the translateral view during surgery. For group A treated with a single plate (LCP; locking compression plate), the operation was performed through a lateral single incision and the fracture site was exposed. The fractures were reduced sequentially and a Kirschner wire was used for temporary fixation. A locking plate with an aiming sight was set under the muscle along the femur surface. After temporary fixation with Kirschner wire at the distal end, a guide pin was drilled at the proximal end of the fracture site to maintain the fracture and plate position. For group B, in which double plates were applied, a single midline incision of the knee joint was made and a rolled towel was placed under the knee to produce 20°–30° of knee flexion. Moreover, a longitudinal incision of the knee joint articular capsule and quadriceps tendon of the patellar side was made, and the distal incision of the articular capsule was extended to the tibial surface direction to dislocate the patella inward. Meanwhile, the incision of the quadriceps tendon was extended toward the proximal part according to the size of the fracture in order to sufficiently expose the fracture part for reduction and to easily locate the medial and lateral metal plates. The extensor retinaculum was preserved over the front of the patella. In difficult situations in which the exposure of the distal femur is not adequate, tibial tubercle osteotomy can be performed (Fig. 2). In some cases, an additional incision was made on the lateral side for proximal fixation of the lateral metal plate. The state of manual reduction with traction was determined using C-arm fluoroscopy during surgery. When it was judged that an anatomical reduction had been obtained, the lateral metal plate (LCP) was first placed on the lateral side temporarily using a K-wire or a reduction retainer and the metal plate (TomoFix-medial distal femur plate; TomoFix-MDF) is thereafter fixed on the medial side. Lateral metal plates were fixed with four or more screws at the distal part and three or more screws at the proximal part according to the fracture line (Fig. 3). For bone defects greater than 1 cm, bone grafting was performed using DBM and allogenic bone chips. In both groups, the operation time, intraoperative bleeding, and amount of transfusion were recorded during surgery.

Rehabilitation started with quadriceps exercise and 30° flexion exercises on the first day after surgery. Wheelchair walking and active knee flexion exercises were encouraged on the fifth day after surgery, and walking with partial weight bearing using a walker was allowed from 1 month after surgery. Walking with full weight bearing was allowed according to the imaging results and clinical findings.

### Assessment methods

The clinical results were assessed using the range of motion (ROM) of the knee joint and the Lysholm knee score with the subjective symptoms and satisfaction of the patient based on a pain-free state during walking with partial weight bearing or when an external force was applied during the final outpatient observation [14]. Functional evaluation was performed using the Knee society score [15]. In both groups, the complications were recorded.

**Fig. 4:**
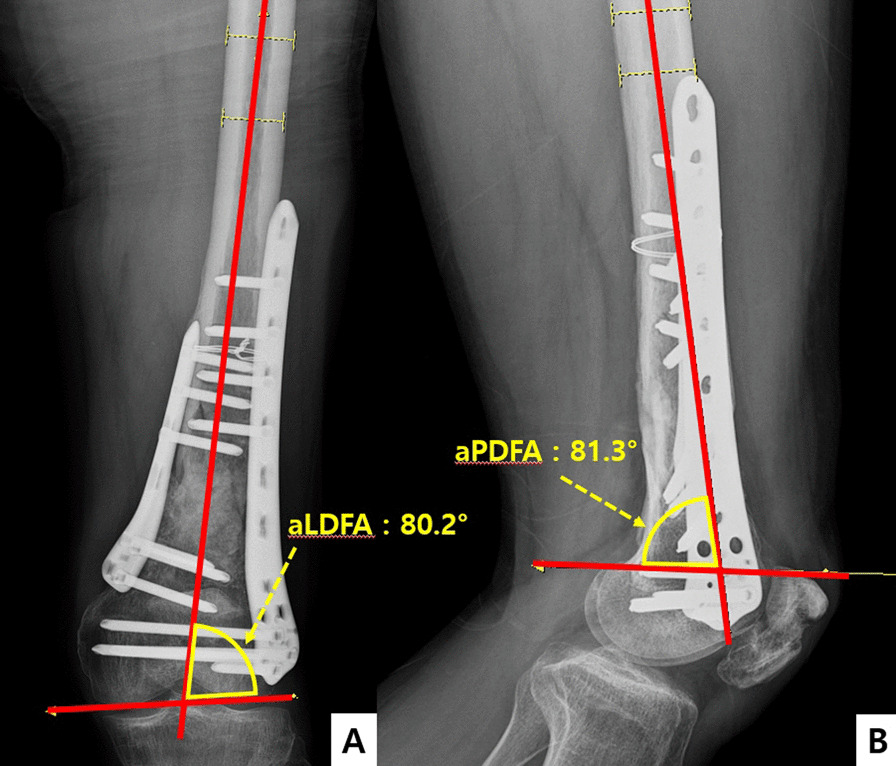
Measurement of aLDFA (**A**) and aPDFA (**B**)

The radiologic results were examined using anterior–posterior radiography of the distal femur and lateral and oblique radiography at 3, 6, 12, 24 weeks, 1 year, and last follow-up, even after bone union was confirmed. Bone union was determined to have been achieved when tenderness of the fracture site was lost, a callus was sufficiently formed, and the trabeculae were connected through the fracture line. The modified radiographic union scale for tibia(RUST) score was used to confirm the objective values as well as the period for these bone unions [16]. For each patient, anatomic lateral distal femoral angle (aLDFA) (Fig. 4a) and anatomic posterior distal femoral angle (aPDFA) (Fig. 4b) were measured on their last available radiograph [17]. Normal values for these parameters were accepted as 81° (range 79°–83°) and 83° (range 79°–87°) respectively. Statistical analysis of preoperative and postoperative clinical results was performed using Student’s t-test with SPSS version 12.0 (SPSS Inc., Chicago, IL, USA).

## Results

The mean age of the patients was 77.3 years (67–87 years) in group A and 76.8 years (64–86 years) in group B, with 18 men and 24 women in group A and 15 men and 25 women in group B. The mean follow-up period was 14.7 months (12–21 months) in Group A and 15.3 (12–26 months) in Group B. The mean bone mineral density of the study group was measured as the T-score: − 3.0 (− 1.2 to − 4.8) in group A and − 3.1 (− 0.6 to − 4.7) in group B (Table 1). There were 18 cases (33-A3) in group B only; type B was a fracture with partial articular surface involvement, with 11 cases (33-B2;10, 33-B3;1) in group A only; and type C was a fracture with complete articular surface involvement, and with a total of 31 cases (33-C1;9, 33-C2;19, 33-C3;3) in group A and 22 cases (33-C1;3, 33-C2;12, 33-C3;7) in group B (Table 2).

**Table 1:** Distribution of patient, age, gender, follow-up and BMI

Variable	Single plate group Group A	Double plate group Group B
Patients Age (years)	42 77.3 (67–87)	40 76.8 (64–86)
Gender		
Female	24 (57.1%)	25 (62.5%)
Male	18 (42.9%)	15 (37.5%)
Follow up (months)	14.7 (12–21)	15.3 (12–26)
BMD T-score	− 3.0 (− 1.2 to − 4.8)	− 3.1 (− 0.6 to − 4.7)

BMD. Bone mineral density

**Table 2:** The fracture types of patients, from single-plate and double-plate group

Variable	Single plate group Group A (*n* = 42)	Double plate group Group B (*n* = 40)
Fracture type (AO/OTA)		
A1/A2/A3	0/0/0	0/0/18
B1/B2/B3	0/10/1	0/0/0
C1/C2/C3	9/19/3	3/12/7

AO/OTA. The AO Foundation/Orthopedic Trauma Association

The average operation time was 81 min (66–92 min) in group A and 110 min (95–120 min) in group B (*p* = 0.33). The mean intraoperative bleeding was 467 ml (338–581 ml) in group A and 573 ml (381–657 ml) in group B (*p* = 0.29). The mean transfusion unit was 0.81 (0 ~ 3) in group A and 1.12 (0 ~ 3) in group B (*p* = 0.47). The average bone union time confirmed by imaging findings was 14 weeks (9–19 weeks) in group A; however, in group B, the average time from bone fracture to bone union was 12.2 weeks (8–19 weeks), except in one case that required bone grafting because of nonunion, with no significant difference between groups (*p* = 0.63). And when the modified RUST score was confirmed as a result of the objective evaluation of the bone union, it was 9.8 points (6–14 points) in group A and 10.4 points (7–14 points) in group B (*p* = 0.38) (Table 3).

**Table 3:** The operation time, intraoperative bleeding, transfusion, bone union period, modified RUST score from single-plate and double-plate group

	Single plate group Group A (*N* = 42)	Double plate group Group B (*N* = 40)	*P* value
Operation time (minutes)	81 (66–92)	110 (95–120)	0.33
Intraoperative bleeding (ml)	467 (338–581)	573 (381–657)	0.29
Transfusion (unit)	0.81 (0–3)	1.12 (0–3)	0.47
Union time (weeks)	14.0 (8–19)	12.2 (9–19)	0.63
Modified RUST score (points)	9.8 (6–14)	10.4 (7–14)	0.38

RUST. radiographic union scale for tibia

The mean ROM of the knee joint, which was examined after bone union was achieved, was 105.0° (flexion contracture 5° and further flexion 127°) in group A and 110.7° (flexion contracture 4° and further flexion 131°) in group B (*p* = 0.37). Between the two groups, aLDFA and aPDFA showed no statistical difference. The average Lysholm knee score was 63.6 points (50–72 points) in group A and 67.1 points (57–75 points) in group B (*p* = 0.44). The average Knee society score was 84.2 points (71–94 points) in group A and 82.9 points (68–96 points) in group B (*p* = 0.53). As the complications of both groups, 1 case of metal failure was confirmed in group A, revision operation was performed, irrigation and debridement were performed on wound infection in 8 cases. 1 case of non-union was confirmed in group B, so autobone graft was performed through revision operation and irrigation and debridement were performed for 2 cases of wound infection (Table 4).

**Table 4:** Clinical and radiologic results and complications from single-plate and double-plate group

	Single plate group Group A (*N* = 42)	Double plate group Group B (*N* = 40)	*P *value
Last follow up knee ROM			
Flexion contracture	5° (2°–10°)	4° (2°–6°)	
Further flexion	127° (95°–135°)	131° (94°–136°)	
ROM (FF-FC)	105° (90°–125°)	110.7° (90°–130°)	0.37
aLDFA	84.7° (81.7°–88.2°)	83.2° (81.3°–86.4°)	0.24
aPDFA	85.3° (81.5°–89.8°)	84.4° (82.6°–87.7°)	0.47
Lysholm knee score	63.62 (50–72)	67.1 (57–75)	0.44
Knee society score	84.2 (71–94)	82.9 (68–96)	0.53
Complications (total)	9 (21.4%)	3 (7.5%)	
Non-union	0	1 (2.5%)	
Metal failure	1 (2.4%)	0	
Wound infection	8 (19%)	2 (5%)	

ROM. Range of motion; FF-FC, further flexion-flexion contracture; aLDFA, anatomic lateral distal femoral angle; aPDFA, anatomic posterior distal femoral angle

All patients with complications showed good clinical results without any specific findings at the last follow up. There was no statistical significance between the two groups in the clinical and radiological results.

## Discussion

With the increasing number of the elderly population, the rate of osteoporotic fractures in elderly patients is also increasing, and studies on the prevention and treatment of these fractures are ongoing. Osteoporotic fractures can easily occur in the elderly, even with low-energy mechanisms. Further, bone healing and bone remodeling are reduced in elderly patients, thereby leading to reduced bone fixation and fusion ability. In addition, fractures lead to exacerbation of the underlying disease in the elderly, resulting in increased mortality [18, 19]. Therefore, a technique using a minimally invasive locking metal plate has been introduced to achieve good treatment results. However, nonunion and fixation failure owing to a lack of bone support for comminuted fractures on the medial side have been reported, and recommendations on the correct method are still lacking.

The treatment of supracondylar fractures of the femur is still challenging for orthopedic surgeons. Surgery for a fracture requires consideration of various factors, such as the patient’s age, type of tools used during surgery, degree of wound damage, degree of intrusion into the joint and femoral shaft, degree of comminuted fracture of the bone, size of the small bone fragment, and degree of bone quality. Intramedullary nails and side plates are being designed to increase stability and limit the movement of the fracture site; however, despite these continuous developments, many failed cases of surgical treatment have been reported because they were applied to patients with poor bone quality or did not provide sufficient stability to withstand the load on distal fractures. Metal failure is believed to be caused by increased interaction of the metal plates and intramedullary nails with increased load at the fracture site. Considering the frequent occurrence of metal failure or varus collapse, we evaluated and compared in this study the single-plate technology and a double-plate technology that includes the medial and lateral sides of the fracture site as treatment options for femoral supracondylar fractures. The application of double plates was believed to decrease the distance between the centers of stress (lever arms) acting on the axis of the femur, thereby decreasing the magnitude of stress acting on the fracture site. In this study, among all patients in group B in whom the double-plate method was applied, all fractures, except for one case that required bone grafting because of nonunion, achieved imaging-confirmed union within an average of 14 weeks. Two cases needed irrigation and debridement of the wound after surgery because of wound infection.

Recently published studies reported a bone union rate of 81–95% with the application of the lateral locking metal plate and a rate of approximately 91% with the application of intramedullary nailing [3, 4, 20–23]. With respect to complications related to internal fixation, metal failure (loosening, breakage) and poor rotation (rotational malposition) were reported to occur in 5–7% and approximately 19–23% of cases, respectively, which require reoperation [3, 22, 23]. Decreased mechanical stability related to the position and length of the metal plate may also emerge as a problem; however, when applying double plates, it is possible to correct the technical error that may occur during surgery by increasing the mechanical stability of the fixation [13]. In our study, in the group using the double plate, LCP was used for the lateral side and tomofix-MDF was used for the medial side. Locked plating is one of the best and modern options for treating supracondylar femur fractures with relatively low failure rates [13]. LCP is a thicker and stiffer plate that combines a conventional hole with a threaded hole in the shaft. This combination allows the use of a conventional screw or a locking screw in the shaft. This mechanism is advantageous because standard screws can be used if bone quality is good or if faced with comminuted fractures or osteoporotic bone locking screws can be used to increase implant stiffness [24]. The Tomofix-MDF plate has a more anatomical pre-formed shape resulting in an improved fit to the distal femur which reduces the distance of the plate to the bone, subsequently reducing leverage forces resulting on the plate. It has an optimized screw-hole orientation, providing a better fit for the screws in the femur. It has also been made slightly shorter and slimmer in favor of reduced prominence [25]. So, it is thought that the surgical treatment using a double plate obtained good results in obtaining stable bone union for fracture in elderly patients or severe metaphyseal communition in our study.

In the treatment of type C3 distal femoral fractures, the advantages of the application of double-plate fixation have been reported in several studies [26–28]. Sanders et al. [26] used a nonlocking condylar buttress metal plate on the lateral side and another metal plate on the medial side with bone grafting in the treatment of nine patients. Ziran et al. [27] used an anterior longitudinal incision to minimize delamination in the medial side of the femur and placed the two metal plates at right angles to each other, and 24 of their 36 patients recovered within approximately 16 weeks. Khalil and Ayoub [28] used a modified Olerud extensile approach to apply double plates to 25 patients, and reported that it took approximately 18.3 weeks to obtain bone union confirmed on imaging.

The knee ROM at follow-up differed between groups in this study, with an average ROM of 110° in the double-plating group and 105° in the single-plating group. We cannot conclude that the double-plating group had a good outcome because the difference in the ROM values was small, and most of the recorded values were estimates obtained by the clinician during follow-up visits (i.e., precise measurements were not required). However, no patient needed reoperation for manipulative correction. A prior study has identified an extensile surgical approach as a risk factor for knee stiffness [28]. However, our procedure involved a median incision, thus minimizing soft tissue damage, and none of the patients experienced knee stiffness. We believe that it is important to allow early ROM in these patients to prevent potential knee stiffness. Therefore, rigid and adequate fixation is needed for these cases.

Rongbin et al. [29] compared a group treated with double plates (group A) and a group treated with a single laterally locking metal plate (group B) for distal femur comminuted fractures. Group A had 0 nonunion cases and group B had two cases of nonunion. Good results of bone union were achieved, and better results were obtained in terms of the Hospital for Special Surgery score. Mohamed et al. [30] treated 16 cases of distal femoral fractures of type C3 using a double-plate fixation technique through an anterior approach, and reported obtaining bone union at an average of 6 months in all cases, with good results in 11 cases. These findings suggest that after solid fixation, facilitating rehabilitation treatment can prevent systemic disease complications associated with osteoporosis fractures in the elderly. Michael et al. [31] performed additional fixation of bone grafts and medial metal plates in 23 cases of nonunion using a lateral metal plate for distal femoral fractures and obtained bone union in 21 cases, and this study may be helpful in the treatment and prevention of nonunion.

The process of approaching the distal femur from the medial side for surgical treatment poses a risk of vascular damage. Computed tomography angiography shows that the blood supply of the medial side of the distal femur is derived from two blood vessels, the medial superior genicular artery and the third perforating artery to the vastus medialis muscle, and it is possible to prevent vascular damage through a careful peeling process because they are not adjacent to the bone. Therefore, the medial incision should be performed approximately 5–7 cm in the vertical proximal direction from the upper part of the distal femur medial condyle, with the two proximal metal screws directly inserted into the femur shaft and the two distal metal screws directly inserted into the distal femur medial condyle [13]. However, as this study showed, the use of an existing incision method for knee replacement surgery has the advantage of reduced internal vascular damage.

One of the complications that can occur after surgical treatment is wound infection. It is one of the complications associated with an open reduction technique, especially in the presence of risk factors such as open fractures, diabetes, and obesity. In addition, a smoking history can also increase the risk of postinjury wound infection [32]. However, no study has reported an increased infection rate with the application of double plates. In this study, postoperative wound infection occurred in eight cases in the operation using a single plate and in two cases in the operation using double plates, which were managed with wound irrigation and debridement. This indicates that risk factors other than the difference in the surgical method caused the wound infection. Therefore, in patients with risk factors for postoperative infection, close observation of wounds after surgery is required. Moreover, encouraging smoking cessation in patients with a smoking history may help prevent wound infections [32].

According to the results of our study, no statistical significance was found in the clinical and radiologic results when a single plate was applied through the lateral incision and when a double plate was applied through the anterior incision. However, single plating, in which only one side is fixed, has disadvantages in that it is difficult to fix fracture fragments and graft for bone defects in elderly patients or severe metaphyseal communition. In these cases, double plating through single incision is one of the useful surgical options that can enable early knee manipulation through anatomical reduction and stable fixation.

This study had some limitations, including its retrospective design, short follow-up period precluding long-term evaluation, and relatively small sample size preventing the generalization of the results.

## Conclusions

In the case of severe distal femur supracondylar fractures in elderly patients, double plating is a useful surgical treatment method because it is relatively easy to expose operation field and enables early knee manipulation through anatomical reduction and stable medial and lateral fixation. Further studies and clinical trials are needed to further investigate the significance of this method.

## Data Availability

The data set supporting the conclusion of this article is available on request to the corresponding author.
